# Induction of thrombospondin-1 partially mediates the anti-angiogenic activity of dexrazoxane

**DOI:** 10.1038/sj.bjc.6605203

**Published:** 2009-09-08

**Authors:** S L Maloney, D C Sullivan, S Suchting, J M J Herbert, E M Rabai, Z Nagy, J Barker, S Sundar, R Bicknell

**Affiliations:** 1 Cancer Research UK Angiogenesis Group, Institute for Biomedical Research, College of Medicine and Dentistry, University of Birmingham, Edgbaston, Birmingham B15 2TT, UK; 2 Molecular Angiogenesis Laboratory, Cancer Research UK, Weatherall Institute of Molecular Medicine, University of Oxford, John Radcliffe Hospital, Oxford OX3 9DS, UK; 3 School of Clinical and Experimental Medicine, College of Medical and Dental Sciences, University of Birmingham, Edgbaston, Birmingham B15 2TT, UK; 4 St John's Institute of Dermatology, King's College, London, UK; 5 School of Cancer Sciences, College of Medicine and Dentistry, University of Birmingham, Edgbaston, Birmingham B15 2TT, UK

**Keywords:** angiogenesis, cell cycle, endothelial, siRNA, pre-clinical

## Abstract

**Background:**

Considerable interest lies in the identification of novel anti-angiogenic compounds for cancer therapy. We have investigated whether dexrazoxane has anti-angiogenic properties and if so, the mechanism of the inhibition.

**Methods:**

The phenotypic effects of dexrazoxane on endothelial cell behaviour was investigated both *in vitro* using human umbilical vein endothelial cells (HUVECs) in cell proliferation, migration, cell cycle and aortic ring assays; and *in vivo* using the mouse angiogenesis subcutaneous sponge assay. Custom angiogenesis pathway microarrays were used to identify differentially expressed genes in endothelial cells after treatment with dexrazoxane *vs* a control. The differentially expressed genes were validated using real-time RT–PCR and western blotting; and the functional effect of one induced gene was confirmed using siRNA technology.

**Results:**

Treatment of endothelial cells with dexrazoxane resulted in a dose–response inhibition of cell growth lasting for up to 5 days after a single dose of the drug. Dexrazoxane was inhibitory in the aortic ring tube forming assay and strongly anti-angiogenic *in vivo* in the rodent subcutaneous sponge model. The anti-angiogenic effect in the sponge was seen after systemic injection into the tail vein as well as after direct injection of dexrazoxane into the sponge. Treatment of microvascular endothelial cells *in vitro* with subtoxic doses of dexrazoxane stimulated thrombospondin-1 (THBS-1) secretion. Knockdown of THBS-1 with siRNA removed the angiogenesis inhibition effect of dexrazoxane, which is consistent with the anti-angiogenic and vascular normalising properties of the drug being principally mediated by THBS-1.

**Conclusion:**

We show that dexrazoxane administered in small repeated doses is strongly anti-angiogenic and that this activity is mediated by induction of the anti-angiogenic THBS-1 in endothelial cells.

Recent studies have shown that anti-angiogenic agents are more effective when given in combination with conventional chemotherapeutic agents rather than as single agents and there is much interest in the identification of novel anti-angiogenic agents (Blumenschein, 2008). The bisdioxopiperazine drug, razoxane (ICRF-159) was originally disclosed as an anti-cancer agent in the 1960s (Herman *et al*, 1982). Razoxane is a racemic drug, with the S-(+)-enantiomer, dexrazoxane (ICRF-187, Zinecard) being more water soluble (Supino, 1984). Early studies with razoxane showed that the drug could inhibit metastasis in the Lewis lung carcinoma model, where the treated tumours had more ‘normalised’ blood vessels compared with the network of poorly defined vascular channels found in control tumours (Hellmann and Burrage, 1969; Salsbury *et al*, 1970, 1974; Le Serve and Hellmann, 1972; Hellmann *et al*, 1974). Subsequent clinical trials of razoxane demonstrated modest anti-tumour activity as a single agent in head and neck carcinomas, acute leukemias, lymphomas and advanced colorectal carcinomas (Gilbert, 1986; Wexler, 1998).

Razoxane also demonstrated activity in combination with radiotherapy against liver metastases from colorectal, inoperable nonmetastatic rectal, bladder, vulval and lung carcinomas; soft tissue and osteosarcomas; and central nervous system tumours including malignant glioma and astrocytoma (Hellmann and Rhomberg, 1991). However, the use of razoxane as an anti-cancer agent was discontinued after a small increase in acute leukaemia with prolonged use of >1 year. More recently, razoxane as an orally administered single anti-angiogenic agent was demonstrated in patients with inoperable renal cell cancer to be well tolerated and resulted in acceptable response rates with very low toxicity and minimal side effects, principally nausea and vomiting (Braybrooke *et al*, 2000).

In current clinical practice, dexrazoxane is used in adjuvant therapy for cardioprotection during anthracycline therapy. It is administered in 3 weekly cycles intravenously at a ratio of multiples, for example, 10 : 1 to anthracycline dosage. At this dosage, it has not been associated with an increased incidence of secondary malignant neoplasms (Barry *et al*, 2008). It is also currently the only antidote for accidental anthracycline extravasation to prevent tissue necrosis (Langer *et al*, 2007).

Dexrazoxane has several biological modes of action. Firstly, it undergoes full ring hydrolysis to form the EDTA analogue, ADR-925, which acts as a chelating agent for divalent cations. The clinical use of anthracyclines is limited by a cumulative cardiotoxicity and this is thought to be due to iron-based oxygen free radical induced oxidative stress on the heart muscle that is low in anti-oxidant enzymes (Hasinoff *et al*, 1998). Chelation of divalent cations is thought to be responsible for the clinical effectiveness of dexrazoxane in reducing the cardiotoxicity of doxorubicin and other anthracyclines. The cardioprotective effect of dexrazoxane is due to blocking of the formation of these reactive oxygen radicals by chelation of unbound transition metals (Huang *et al*, 1982; Synold *et al*, 1998). Secondly, dexrazoxane, but not ADR-925, displays strong DNA topoisomerase (topo) II inhibitory activity (Hasinoff *et al*, 1995). The topo II inhibition exerted by dexrazoxane is due to its binding and stabilisation of the protein/DNA complex. This is distinct from etoposide, which acts by inhibiting topo II mediated resealing of DNA strand breaks and aclarubicin, which inhibits the binding of topo II to its DNA substrate.

The anti-tumour activity of razoxane and dexrazoxane may therefore be due to iron chelation (Creighton *et al*, 1969), inhibition of the enzymatic activity of topo II (Tanabe *et al*, 1991), a combination of both or some other unknown mechanism.

It has recently been demonstrated that anti-angiogenic agents are likely to work through a process of normalisation of vasculature wherein leaky tortuous vessels in tumours are ‘normalised’ and excess vessels pruned, resulting in a reduction of interstitial fluid pressure during a transient ‘normalisation window’ (Tong *et al*, 2004; Willett *et al*, 2004). This normalisation of the tumour vasculature increases blood flow and oxygen to the tumour. Delivery of chemotherapeutic drugs during that window improves cytotoxic drug delivery resulting in tumour response and improved survival. Indeed, this is the mechanism of action of ‘Bevazicumab’ the first anti-angiogenic agent to show improvement in survival in colorectal cancer when co-administered with cytotoxic agents but has limited anti-tumour activity on its own (Hurwitz *et al*, 2004). The early studies showing that razoxane is anti-metastatic with accompanying normalisation of blood vessels in tumours suggests that razoxane could have anti-angiogenic properties, but to date this has not been documented.

Given the interest in vascular normalising agents, we investigated whether dexrazoxane was anti-angiogenic and whether the anti-angiogenic properties of the drug could be explained by its known modes of action or whether there existed some other as yet undiscovered mechanism of action. We show that dexrazoxane administered in small repeated doses is strongly anti-angiogenic and that this activity is mediated by induction of the anti-angiogenic THBS-1 in endothelial cells.

## Materials and methods

### Endothelial cell growth response curves and proliferation assays

Human dermal microvascular endothelial cells (HDMECs, Clonetics, San Diego, CA, USA) and human umbilical vein endothelial cells (HUVECs)—isolated as per protocol (Jaffe *et al*, 1973) were seeded either into six-well plates at 50 000 cells per well and into 96-well plates at 2000 cells per well and allowed to attach for 24 h. The cells were treated with drug, and cell numbers were counted after the desired incubation time using a Beckmann Coulter counter. Additionally, cell numbers were determined by MTS assay (Promega, Madison, WI, USA). Briefly, the MTS dye, 3-(4,5-dimethylthiazol-2-yl)-5-(3-carboxymethoxymethoxyphenyl)-2-(4-sulfo-phenyl)-2H-tetrazolium, is converted to a formazan product that is soluble in tissue culture medium and quantified by its absorbance at 492 nm. The cell number was proportional to MTS dye conversion within the range used in these experiments.

### Migration assay

Analysis of HUVEC migration was performed using the Angiogenesis Endothelial Cell Migration Kit (BD BioCoat, BD Bioscience, San Jose, CA, USA). The 5 × 10^4^ HUVEC cells per well were seeded into the upper chamber of the migration plate insert in migration media (MCDB 131, 1% FCS and 5 IU ml^−1^ heparin). The lower chambers contained 750 *μ*l of 10 ng ml^−1^ vegf121. After 22 h incubation at 37°C, the insert membranes were stained with 4 *μ*g ml^−1^ Calcein AM (Molecular Probes, Invitrogen, Paisley, UK) in Hanks Balanced Salt Solution for 90 min. Fluorescence on the underside of the membrane was measured at excitation/emission wavelengths of 485 out of 530 nm. Images were taken using a Zeiss Axiovert 135 microscope with an Axiocam digital camera at 10 × magnification.

### Cell cycle analysis

The 1500 HUVEC cells were seeded on to 0.1% gelatine coated well of a 96-well plate in replicates of eight and incubated at 37°C/5% CO_2_ overnight to adhere. The following day and at subsequent 24-h intervals, the media was replaced with media containing 50 *μ*M dexrazoxane. At the end of the time course, media was carefully removed by pipette from all wells and cells fixed in 85% ice-cold ethanol. This was later removed by pipette and cells were carefully washed with PBS, which was again carefully removed by pipette. Cell were incubated in the dark at 37°C for 15 mins post-RNase A treatment and made permeable with triton X before staining with PI. The 96-well plates were subsequently scanned using the Acumen ^e^X3 machine (TTP LabTech, Melbourn, Royston, UK).

### Rat aortic ring assay

Thoracic aortas were harvested from 6- to 8-week-old rats on the morning of the assay. Matrigel that had been thawed at 4°C overnight was diluted 1 : 1 with PBS. A measure of 110 *μ*l was then aliquoted per well into a 48-well plate and the plate was incubated at 37°C for 30 min to allow the matrigel to set. The aorta was rinsed in endothelial cell medium and any fibro-adipose tissue was removed. The aorta was cut into 1–1.5 mm rings using a scalpel blade. The aortic rings were placed on top of the matrigel in the centre of the well, a further 40 *μ*l of matrigel was added to hold the aorta in place, and the plate was returned to the incubator for a further 30 min. Growth medium was then added carefully to a volume of 200 *μ*l per well. Drug was added 24 h later and the extent of angiogenesis was assessed 5–6 days later.

### Mouse sponge assay

Sterile 8-mm polyurethane sponge discs were inserted under the dorsal skin of anaesthetised black C57 female mice. Dexrazoxane (30 mg kg^−1^) with bFGF (5 mg kg^−1^) in 0.5% carboxymethylcellulose in saline (carrier solution) was injected into the sponges on days 1, 3, 5, 7, 9, 11, 13, 15 and 17 after sponge implantation. Control groups received bFGF in carrier solution or carrier solution alone. A second experiment involved injection of dexrazoxane (30 mg kg^−1^) into the tail vein on days 2, 6, 10, 14 and 18 preceded by injection of bFGF (5 mg kg^−1^) into the sponge on days 1, 5, 9, 13 and 17 after sponge implantation. Mice were sacrificed on day 21, and sponges with surrounding tissue were excised and fixed in 3.5% paraformaldehyde. All *in vivo* experiments were performed in accord with British Home Office license number PPL 70/4949. All animal experiments were conducted at Clare Hall animal laboratories, Cancer Research UK and all procedures were within local institute and national ethical guidelines and were in compliance with the United Kingdom coordinating committee on cancer research guidelines for the welfare of animals in experimental neoplasia.

### Angiogenesis pathway arrays

Nitrocellulose membrane gene arrays (GEArray Kit, Super Array Inc, Bethesda, MD, USA) were used to determine the effect of dexrazoxane on the regulation of genes involved in angiogenesis following manufacturer's instructions. Briefly, 32P-labelled cDNA probes were synthesised from total RNA from untreated HDMEC and from HDMEC that had been treated with 50 *μ*M Dexrazoxane for 24 h. The cDNA probes were hybridised to gene-specific cDNA fragments spotted on the membranes. The relative expression levels of the genes on the array were determined using a phosphorimager and quantitation software.

### Western blot analysis

After treatment with Dexrazoxane or THBS-1 siRNA, aliquots of the medium were collected and the cells were washed with PBS and lysed in lysis buffer to obtain total protein extract. Western blotting was performed using 10 *μ*g of whole cell lysates and antibodies to THBS-1 (Neomarkers, MA, USA). Immunodetection of *β*-tubulin and MCM7 were used as loading controls.

### Transfections with siRNA

A measure of 10^6^ HUVECs were seeded on a 0.1% gelatine coated 10 cm plate the day before transfection. siRNA duplexes generated were THBS-1 siRNA1 (5′-GGAGTTCAGTACAGAAATA-3′), THBS-1 siRNA 2 (5′-GTACAGAAATAACGAGGAA-3′) and negative control duplex (Eurogentec, Hampshire, UK). Transfection of HUVEC was carried out using 50 nmol l^−1^ of siRNA duplexes with 0.3% lipofectamine RNAi Max in optiMEM (Invitrogen, Paisley, UK). Cells were incubated for 5 h at 37°C/5% CO_2_ then the mix was replaced with medium without antibiotics. At 24 and 48 h posttransfection, knockdown was assessed by RT–PCR, real-time PCR and western blot.

### RT–PCR

Total RNA was extracted from HUVEC using the RNeasy mini kit (Qiagen, Crawley, West Sussex, UK) according to the manufacturer's protocol. Complementary DNA was prepared using 1 *μ*g of total RNA and the random priming High-Capacity cDNA Archive kit (Applied Biosystems, Foster City, CA, USA) according to the manufacturer's protocol. The primers THBS-1 mRNA Forward (5′-TTGTCTTTGGAACCACACCA-3′) and THBS-1 mRNA Reverse (5′-CTGGACAGCTCATCACAGGA-3′) were used at 2.5 pmol with 12.5 *μ*l of 2xGoTaq green master mix (Promega, Madison, WI, USA). Reaction conditions were 95°C for 5 min; 95°C for 45 s, 60°C for 1 min, 72°C for 2 min; repeated for 30 cycles; 72°C for 10 min. Agarose gel electrophoresis gave rise to a 187-bp product when ran on a 2% agarose gel according to standard protocols.

### Real-time PCR

RNA and cDNA were prepared as above and reaction conditions optimised for the Rotor-Gene 3000 real-time DNA analysis system (Corbett Research, Sydney, Australia) to obtain the best amplification kinetics. The 25-*μ*l reaction mix contained 12.5 *μ* of 2 × Sensimix, 1 *μ*l of 10 *μ*M Sense primer, 1 *μ*l of 10 *μ*M anti-sense primer and 0.25 *μ*l of 10 M probe, with cDNA dilution replicates of 1 : 10, 1 : 100 and 1 : 1000; water was used as a no template control. Cycling conditions were 95°C for 10 mins followed by 40 cycles of 95°C for 10 s, 58°C for 15 s, 72°C for 15 s, 82°C for 15 s. Standard curve analysis was performed to obtain relative expression levels for THBS-1 and the housekeeping gene *β*-actin to which THBS-1 expression was normalised. The cycle threshold was determined from a curve generated from a plot of cycle number *vs* fluorescence with a manual threshold set above the background fluorescence of the no template control. The dCT method was used to calculate relative gene expression levels as per protocol by applied biosytems. The levels of expression of the interferon (INF) response genes, IGS 20 and OAS I were determined as described after siRNA treatment.

### Data analysis

All statistical analyses (*t*-tests and ANOVAs) were performed using R and MS Excel functions.

## Results

### Effect of dexrazoxane on endothelial cell behaviour *in vitro*

**Figure 1:**
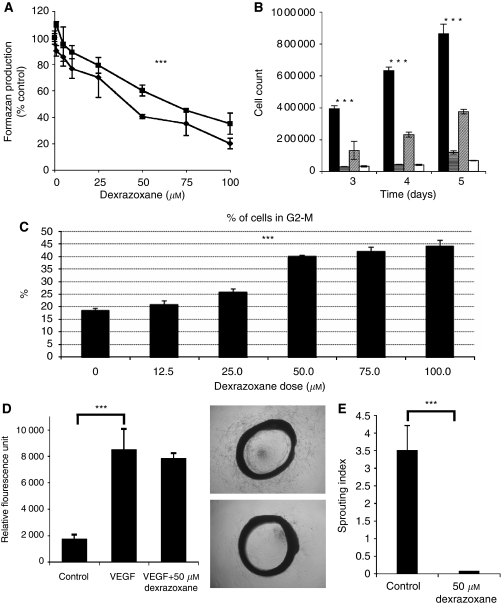
Effect of dexrazoxane on angiogenesis *in vitro*. (**A**) Effect of Dexrazoxane on cell proliferation in endothelial cells. Cell proliferation was measured using the MTS assay 3 days after treatment of HDMEC (–▪–) and HUVEC (⧫) with Dexrazoxane. A one-way ANOVA showed that increasing the concentration of Dexrazoxane statistically significantly reduced cell proliferation (^***^P-value <0.001) in both HUVECs and HDMECs. (**B**) Effect of Dexrazoxane on endothelial cell growth over 5 days. Cell numbers were counted on days 3, 4 and 5 after a single dose of 50 *μ*M Dexrazoxane. The treatment groups were HUVEC control (▪), HUVEC treated with 50 *μ*M Dexrazoxane (
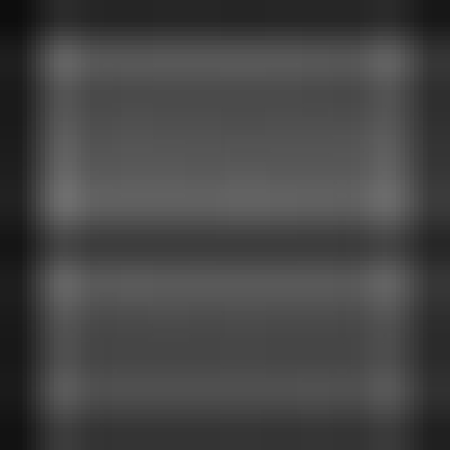
), HDMEC control (
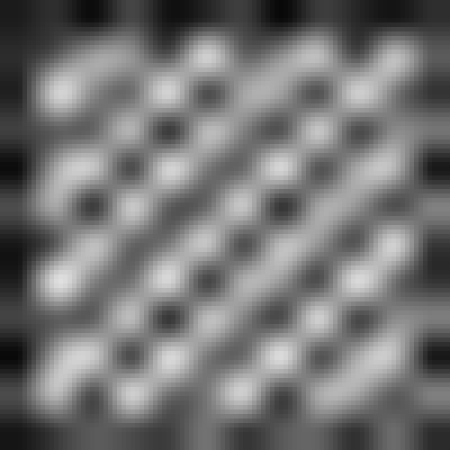
) and HDMEC treated with 50 *μ*M Dexrazoxane (□). Statistical significance of Dexrazoxane treatment *vs* control was determined using a *t*-test, indicated by ^***^ (*P*-value <0.001) in the figure. Results show that Dexrazoxane treatment significantly reduced the number of cells. (**C**) Effect of increasing doses of dexrazoxane on the number of HUVEC in G2M phase of cell cycle. A one-way ANOVA shows that increasing the concentration of dexrazoxane statistically significantly increases the number of cells in G2-M phase with a *P*-value <0.001 (^***^). (**D**) Effect of dexrazoxane on endothelial cell migration. The effect of 50 *μ*M dexrazoxane on VEGF stimulated HUVEC migration was tested *vs* a control. Using a *t*-test, no statistical significance was found between the VEGF alone and VEGF+Dexrazoxane-treated migration of cells. However, in contrast, significant *P*-values of <0.001 (^***^) were found comparing both of these to the untreated control cell migration. (**E**) Effect of dexrazoxane on the aortic ring assay. Aortic ring sprouting was scored on a scale of 0 (no sprouts), 1 (a few sprouts), 2 (intermediate sprouting), 3 (substantial sprouting), 4 (complete sprouting). The sprouting from each ring was assessed by two independent observers and the inter-observer variability was found to be <0.001. A 50-*μ*M dose of Dexrazoxane had a statistically significant effect in reducing the sprouting index of the aortic ring (*t*-test, *P*-value <0.001).

The effect of dexrazoxane on endothelial cell proliferation *in vitro* was examined using HUVEC and HDMEC isolates. Increasing doses of dexrazoxane was found to cause a statistically significant (*P*-value <0.001) decrease in cell proliferation (Figure 1A) with HDMEC being somewhat more sensitive to dexrazoxane than HUVEC (HUVEC IC_50_=71 *μ*M; HDMEC IC_50_=52 *μ*M). The extent of the growth inhibition of dexrazoxane was determined in both HUVEC and HDMEC by treating with a single dose of 50 *μ*M and then counting cell numbers on days 3, 4 and 5 after treatment (Figure 1B). It was found that 50 *μ*M dexrazoxane caused growth inhibition of HDMEC and HUVEC for up to 5 days (*P*-value <0.001). Concurrent with this inhibition, increasing doses of dexraxoxane caused an accumulation of cells in the G2M phase of the cell cycle (*P*-value <0.001), peaking at around 50 *μ*M (Figure 1C).

In contrast to the effect on cell proliferation, dexrazoxane was found to have no effect on VEGF stimulated cell migration (Figure 1D). Daily dosing with dexrazoxane (50 *μ*M) was found to almost completely inhibit sprouting from aortic rings (Figure 1E, *P*-value <0.001). If the medium was changed to dexrazoxane-free medium at any time for up to 8 days after seeding the ring, then sprouting occurred showing that dexrazoxane was having a specific growth inhibitory effect rather than simply a cytotoxic one.

### Effect of dexrazoxane on *in vivo* angiogenesis

**Figure 2:**
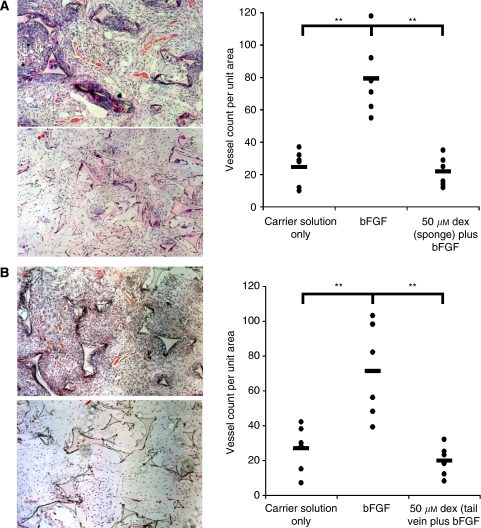
Effect of dexrazoxane *in vivo*. Effect of dexrazoxane on angiogenesis *in vivo*, using the mouse sponge assay. (**A**) The number of vessels per unit area of sponge was counted after injection of carrier solution only, bFGF only and bFGF with 50 *μ*M dexrazoxane. The top left panel shows a typical example of a sponge injected with bFGF alone, whereas the bottom left panel shows a typical example of a sponge injected with bFGF with dexrazoxane. (**B**) The number of vessels per unit area of sponge was counted after injection of 50 *μ*M dexrazoxane into the tail vein on the day after injection of bFGF directly into the sponge. Both sponge assays produced a statistical significant difference between carrier solution control *vs* bFGF (*P*-value <0.01) and bFGF *vs* bFGF with dexrazoxane. No significant difference was found between carrier solution control *vs* bFGF with dexrazoxane.

The effect of dexrazoxane *in vivo* was determined using the mouse sponge assay (Figure 2). Two dosing protocols were tested. The drug was either injected directly into the sponge (Figure 2A) or into the tail vein (Figure 2B). Injection of dexrazoxane into the sponge was found to result in lower overall vessel numbers (*P*-value <0.01), an absence of large vessel and lower cell density when compared with injection of bFGF alone. A similar result was obtained when dexrazoxane was injected into the tail vein (Figure 2B, *P*-value <0.01).

### Dexrazoxane induces THBS-1 expression in HUVEC

**Figure 3:**
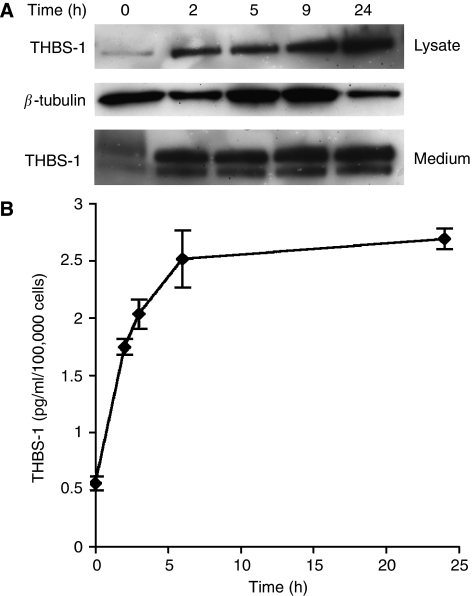
Upregulation of thrombospondin-1 by dexrazoxane *in vitro*. (**A**) Western blotting of HUVEC whole cell lysate and medium after treatment with 50 *μ*M Dexrazoxane for 2, 5, 9 and 24 h displayed an increase in THBS-1 protein expression over the time course. (**B**) This result was confirmed by ELISA using conditioned medium of cells treated with Dexrazoxane at different time points.

Arrays of genes involved in processes relating to angiogenesis were used to screen for differential gene expression after treatment of endothelial cells with 50 *μ*M dexrazoxane. Of most interest in view of its known anti-angiogenic activity was a 3.7-fold upregulation of THBS-1 expression. To confirm this result, cells were treated for 2, 5, 9 and 24 h with 50 *μ*M dexrazoxane, followed by western blotting of the total cell lysate to measure bound THBS-1 protein and the medium to measure secreted THBS-1 protein (Figure 3A). The THBS-1 level in the cell lysates was found to increase with 50 *μ*M dexrazoxane and continued to increase for up to 24 h after drug treatment. The levels of secreted THBS-1 increased sharply 2 h after treatment and showed a plateau when measured by western blotting. This result was confirmed using a human THBS-1 ELISA (Figure 3B). When measured by ELISA, THBS-1 secretion increases for up to 5 h after dexrazoxane treatment and then remained steady for up to 24 h.

**Figure 4:**
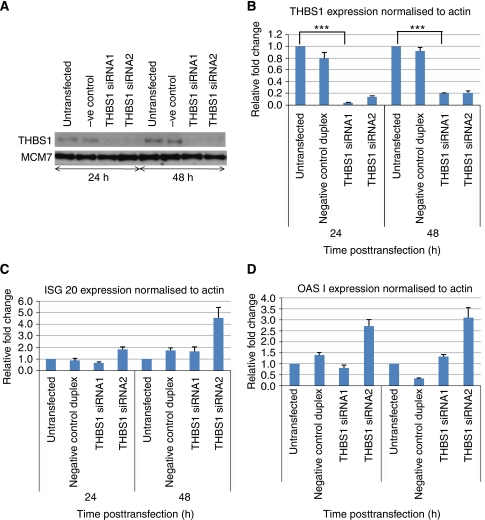
Downregulation of THBS-1 by siRNA. (**A**) Western blot showing the downregulation of THBS-1 in HUVEC at 24 and 48 h posttransfection with THBS-1 targeting siRNA. (**B**) Real-time PCR showing the downregulation (*P*-value <0.001) of THBS-1 in HUVEC, targeted by THBS-1 siRNA at 24 and 48 h posttransfection. (**C** and **D**) Real-time PCR showing no significant upregulation of the interferon response genes ISG 20 and OAS I in HUVEC targeted with THBS-1 siRNA (duplex 1) at 24 and 48 h posttransfection.

We investigated whether THBS-2, which is also anti-angiogenic, could mediate the THBS-1 insensitive inhibition. Analysis of HUVEC mRNA and protein lysates showed that they do not express THBS-2 (data not shown). To determine the contribution of THBS-1 secretion towards dexrazoxane-mediated anti-angiogenesis, THBS-1-specific siRNAs were designed. Western blotting of HUVEC lysates showed that THBS-1 protein was reduced at 24 and 48 h posttreatment with siRNA compared with controls (Figure 4A). RT–PCR of RNA extracted from HUVEC treated with THBS-1 siRNAs showed downregulation of THBS-1 using two specific siRNA duplexes (data not shown). This was confirmed using real-time RT–PCR and showed a 95% knockdown at 24 h and 82% knockdown at 48 h posttransfection with 50 nM siRNA duplex 1 (Figure 4B). This result was sustained on titration down to 12 nM siRNA (data not shown). Levels of THBS-1 started to return to baseline both at the RNA and protein level from 72 h posttransfection with siRNA (data not shown). Introduction of siRNA into cells can give artifactual results attributable to induction of INF secretion from the transfected cell (the so-called INF response) (Reynolds *et al*, 2006). To rule out the possibility of an INF response being generated by siRNA duplexes, the INF response genes ISG20 and OASI were examined by real-time RT–PCR post-siRNA transfection. Duplex 1 at a concentration of 50 nmol l^−1^ failed to illicit an INF response and so this duplex was taken forward in subsequent experiments (Figure 4C and D).

**Figure 5:**
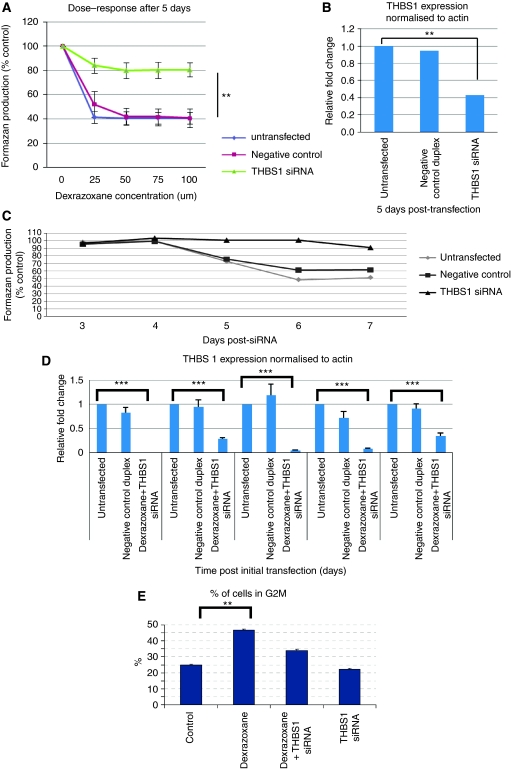
Effect of downregulation of THBS-1 on cell proliferation after treatment with dexrazoxane. (**A**) MTT assay showing cell proliferation 5 days posttransfection with THBS-1 siRNA and dose–response to 50 *μ*M dexrazoxane given at 24-h intervals. The results show that the anti-proliferative effect of dexrazoxane is partially mediated by THBS-1 as its knockdown shows significant increase in cell proliferation (*P*-value <0.01 at 25 *μ*M through to 100 *μ*M) as compared with the untransfected and negative controls. (**B**) Real-time PCR showing statistically significant downregulation (*P*-value <0.01) of THBS-1 in HUVEC treated with THBS-1 siRNA at 5 days posttransfection. Although still significantly differentially expressed (knockeddown) at 5 days posttransfection, the percentage knockdown has decreased by 38% as compared with 24 h. (**C**) MTT assay showing cell proliferation 3–7 days posttransfection with THBS-1 siRNA at day 0 and again at day 3 with concurrent treatment with 50 *μ*M dexrazoxane at 24-h intervals. (**D**) Real-time PCR showing downregulation of THBS-1 in HUVEC from 3 to 7 days with transfection with THBS-1 siRNA taking place at day 0 and again at day 3. The downregulation of THBS-1 was statistically significant at all time points, *P*-value <0.001. (**E**) Proportion of HUVEC in G2M phase of cell cycle 5 days posttransfection with THBS-1 siRNA and treatment with 50 *μ*M dexrazoxane at 24-h intervals. Dexrazoxane treated cells alone *vs* the control showed statistical difference (*P*-value >0.01) in the number of cells in G2M phase. This effect was reversed with THBS-1 knockdown.

To determine the contribution of THBS-1 secretion towards the inhibitory effect of dexrazoxane on angiogenesis (or the anti-angiogenic activity of dexrazoxane), MTS assays were carried out along with real-time RT–PCR after THBS-1 knockdown and treatment with dexrazoxane. This revealed that 5 days post-siRNA transfection and dexrazoxane treatment of HUVEC, there was a 40% decrease in cell proliferation, which was reduced to a 20% decrease after THBS-1 knockdown. However, real-time RT–PCR analysis showed that the level of THBS-1 knockdown at this time point was only 57% compared with controls (Figure 5B). To achieve a greater knockdown, HUVEC were treated with 50 nM THBS-1-specific siRNA and received a further treatment 72 h after the initial transfection when the levels of THBS-1 start to rise. They were further treated with 50 *μ*M dexrazoxane at 24-h intervals for 7 days. At 5 and 6 days posttreatment of HUVEC, there was now a 27.3% decrease and 51.6% decrease, respectively, in cell proliferation compared with controls, an effect which was completely ameliorated by treatment with THBS-1 siRNA (Figure 5C). Thus, knockdown of THBS-1 in dexrazoxane-treated HUVEC completely blocked the effect of the drug on cell proliferation. Levels of THBS-1 knockdown were quantified over the time course by real-time RT–PCR and showed a knockdown of THBS-1 at the RNA level at days 5 and 6 of 96% and 93%, respectively (Figure 5D).

Dexrazoxane increased the number of cells in G2M, presumably by a G2M block. This effect was largely reversed by THBS-1 knockdown, supporting the cell proliferation work (Figure 5E).

## Discussion

Early studies with razoxane showed that it has anti-metastatic and putative blood vessel normalisation properties (Hellmann and Burrage, 1969) and it is possible that it could also have anti-angiogenic activity, although the latter has never been formally shown. We have now performed *in vitro* and *in vivo* studies to show that dexrazoxane is anti-angiogenic and that this action is mediated through upregulation of THBS-1. Dexrazoxane showed clear effects on the vasculature, for example, in the rodent subcutaneous sponge assay dexrazoxane was able to reduce bFGF-induced angiogenesis after direct injection into the sponge or injection into the tail vein with the FGF injected into the sponge.

Dexrazoxane is metabolised *in vivo* to its one ring opened hydrolysis product, ADR-925, which has a structure similar to that of EDTA (Buss and Hasinoff, 1997). ADR-925 is detectable within 5 min of intravenous administration of dexrazoxane to rats, suggesting that dexrazoxane is rapidly metabolised *in vivo* (Schroeder and Hasinoff, 2002). The hydrolysis products of dexrazoxane do not inhibit topo II, unlike dexrazoxane itself (Hasinoff *et al*, 1995). High millimolar concentrations of dexrazoxane hydrolysis products are required *in vitro* for cytotoxicity compared with micromolar concentrations for the parent compound (Hasinoff *et al*, 1995). Therefore, it seems unlikely that the systemic anti-angiogenic effects of dexrazoxane in the rodent sponge assay are due to topo II inhibition by dexrazoxane.

By use of microarray, we found that THBS-1 is markedly upregulated in endothelial cells exposed to dexrazoxane. The increase in mRNA expression correlated with a corresponding increase in THBS-1 protein expression and THBS-1 protein secretion for at least 24 h after drug treatment. To determine whether the observed anti-proliferative properties of dexrazoxane on endothelial cells were due to topo II or increased THBS-1 secretion, we used siRNA to target THBS-1 mRNA in HDMEC and HUVEC. Cells unable to express THBS-1 were not growth inhibited by dexrazoxane consistent with THBS-1 upregulation being a part of the anti-angiogenic mechanism of action of dexrazoxane.

Recent research has suggested that THBS-1 could be a mediator of the anti-angiogenic effects of low-dose metronomic chemotherapy (Bocci *et al*, 2003). Unlike conventional cancer chemotherapy, low-dose metronomic chemotherapy targets dividing endothelial cells in the newly forming blood vessels within tumours rather than the surrounding genetically unstable tumour cells (Browder *et al*, 2000). This has been called anti-angiogenic chemotherapy or metronomic dosing (Hanahan *et al*, 2000). The advantages of this approach are that the lowered drug dose gives fewer side effects, whilst significantly delaying the onset of drug resistance and improving the efficacy and durability of chemotherapy (Browder *et al*, 2000; Klement *et al*, 2000; Colleoni *et al*, 2002). The possible selective nature of this type of chemotherapy towards endothelial cells was reinforced when it was found that exposure to protracted low-dose chemotherapy *in vitro* and *in vivo* resulted in a marked induction of THBS-1 (Bocci *et al*, 2003). In our study, we compared the effect of local injection into the sponge with systemic injection into the tail vein on the blood vessel density of the sponge. The protocol used for the local injection experiment resembled that of a metronomic dosing schedule. The maximum tolerated dose (MTD) of dexrazoxane in mice is 250 mg kg^−1^, 7 times higher than our dose of 35 mg kg^−1^, which was given every second day for 17 days. We propose, therefore, that in small regular doses, administration of dexrazoxane potently inhibits angiogenesis.

Thrombospondins are secreted calcium binding glycoproteins implicated in a number of cellular processes including wound healing and cancer (Tuszynski *et al*, 1987a, 1987b; Asch and Nachman, 1989; Sargiannidou *et al*, 2001).

The family has five members, two of which, THBS-1 and THBS-2 have been shown to play a role in platelet aggregation, wound healing and angiogenesis through their N-terminal properdin type I repeats (Volpert *et al*, 1995; Bonnefoy *et al*, 2008). Expression of THBS-1 has been shown to be up-regulated by hypoxia and heat shock. In contrast, a number of growth factors including TNF*α*, IL-1*β* and a variety of transforming oncogenes such as c-jun, reduce expression (Adams, 1995; Watnick *et al*, 2003). THBS-1 is induced by p53 and binds to CD47, CD36, integrins and heparin sulphate proteoglycans (Sid *et al*, 2004) in turn inhibiting the effects of VEGF, bFGF and IL-8 (Dameron *et al*, 1994a, 1994b). The endothelial cell apoptotic activity of THBS-1 is mediated through interaction with the Fas/Fas ligand, caspase-3 activation and FAK fragmentation (Volpert *et al*, 2002).

There has been significant interest in THBS-1 mimetics as anti-angiogenics, as it is not feasible to administer THBS-1 itself due to its large size. However, peptide mimetics have suffered severe *in vivo* stability problems (Giavazzi and Taraboletti, 1999). The outcome of a phase II study investigating the efficacy of a THBS-1 mimetic (ABT-510) in soft tissue sarcoma showed safety but no compelling evidence for use as a single agent. The authors suggest that further validation in combination with current chemotherapy regimes, such as 5-Flurouracil (5-FU) may be of benefit. The proposed mode of action is to block binding of THBS-1 to endothelial cells, inducing expression of Fas ligand and preventing VEGF- and bFGF-induced migration (Baker *et al*, 2008).

A number of chemotherapeutic agents such as 5-FU and trastuzumab up-regulate THBS-1, with 1 mol l^−1^ of 5-FU up-regulating the level of THBS-1 three-fold in KM12C human colon carcinoma cells and two-fold in HUVEC and human colon adenocarcinoma LOVO cells (Zhao *et al*, 2008). The transcription factor Sp-1 has been shown to be important for the EGF-induced THBS-1 expression (Okamoto *et al*, 2002). Cytotoxic drugs have been shown to up-regulate members of the MAPK family (Srivastava *et al*, 1999) and Zhao *et al* suggest that p38 MAPK signalling functions in 5-FU, trastuzumab and TGF*β*1 regulation of Egr-1and THBS-1.

This work is of particular significance with the advent of anti-vascular endothelial growth factor antibody Bevazucimab (Avastin, Gentec, South Francisco, CA, USA) and the realisation that its mechanism of anti-tumour activity is primarily due to normalisation of tumour vessels (Willett *et al*, 2004). Identification of the mechanism of anti-angiogenic action now identifies thromospondin-1 as a potential surrogate of response and investigation and monitoring of THBS-1-1 levels may be of benefit in future clinical trials with dexrazoxane. Dexrazoxane is a very inexpensive but potent anti-angiogenic agent. In conclusion, our results demonstrate that dexrazoxane when administered in small regular doses acts *in vivo* as a potent anti-angiogenic agent and this action is at least in part mediated by an upregulation of THBS-1.
